# Integrins outside focal adhesions transmit tensions during stable cell adhesion

**DOI:** 10.1038/srep36959

**Published:** 2016-11-15

**Authors:** Yongliang Wang, Xuefeng Wang

**Affiliations:** 1 Department of Physics and Astronomy, Iowa State University, 12 Physics Hall, Ames, IA 50011, USA; 2 Molecular, Cellular, and Developmental Biology interdepartmental program, Molecular Biology Building, Ames, IA 50011, USA

## Abstract

Integrins coupled with other proteins form protein complexes named focal adhesions (FA) which are considered as the primary sites for cellular forces transduction during cell stable adhesion. Cell traction forces transmitted by FAs and integrin tensions inside FAs have been extensively studied. However, it remains unknown whether integrins outside FAs can transmit tension, and if so, what is the tension range. We previously developed a tension sensor named tension gauge tether (TGT). To calibrate integrin tensions outside FAs, here we applied multiplex TGT (mTGT) to simultaneously monitor integrin tensions at separate levels. mTGT unambiguously revealed that integrins outside FAs also transmit tension after FA formation. These tensions are mainly located in the range of 43 ~ 54 pN which is lower than integrin tensions inside FAs. Integrin tensions both inside and outside FAs substantially contribute to bulk cellular forces and they respond independently to actin and myosin II inhibition, serum deprivation and microtubule inhibition, indicating their different tension sources and independent dynamics. Our work identified integrin tensions outside FAs and calibrated the tension range for the first time. We also demonstrated that mTGT is a valuable tool to monitor integrin tension profile in a broad detection range of 10 ~ 60 pN.

Integrins are the major membrane proteins that establish physical linkage and mediate mechanical signaling between cells and the matrix^1^. Integrin tensions are fundamental mechanical signals mediating cell mechano-sensing. After integrins bind to ligands presented on the matrix, cells actively apply forces on integrin-ligand bonds to probe the surrounding environment and regulate cell adhesion, migration, proliferation, differentiation and so on^2^,^3^,^4^. During stable cell adhesion, integrins cluster with many other proteins and form focal adhesions (FA) which are considered as the main transduction sites of physical force and biomechanical signals between cells and the matrix^5^,^6^. Because of the important role in integrin signaling, FA structure and cellular forces transmitted by FAs have been extensively studied^7^,^8^,^9^,^10^. However, it remains unknown whether integrins outside FAs also transmit cellular forces after FA formation. The outside-FA integrin tensions have only been hinted to exist in cells. For example, previous research reported that cell traction forces do not correlate with FA distirbution and integrins in FAs only contribute 30% of adhesion strength^11^. In addition, FA formation is force dependent^12^,^13^, suggesting that outside-FA integrin tensions may exist and stimulate the formation of new FAs. Here we use the term “outside-FA integrin tensions” to denote the tensions transmitted by integrins outside FAs. This term also includes integrin tensions in the potential nascent adhesions if these adhesions are small and unresolvable to the regular fluorescence microscopy. Because the outside-FA integrin tensions likely contribute to cell traction forces and may initiate FA formation, their characterization would contribute to a better understanding of integrin functions. However, so far there was no direct experimental confirmation or calibration of the outside-FA integrin tensions. In recent years, a series of surface-tethered molecular tension sensors have been developed to measure and map integrin tensions in real-time^14^,^15^,^16^,^17^,^18^,^19^. These techniques provide quantitative approaches to study integrin tensions, and revealed new insights to integrin signaling pathways. To date, integrin tension measurements with surface-tethered tensions sensors were mainly conducted in FAs, and outside-FA integrin tensions remain unconfirmed. There are two possible reasons why outside-FA integrin tensions were not calibrated till now: 1. Outside-FA integrin tensions are more dispersive on surface in comparison to integrin tensions concentrated in FAs and that may lead to low or even undetectable signals for real-time tension sensors. 2. Tension sensors referenced above generally have a dynamic range limited in 0 ~ 20 pN which may not be high enough to differentiate FA and outside-FA integrin tensions. Integrin tensions in FAs have been previously shown to be above 54 pN^20^ or even as high as 110 pN^21^. We will show that outside-FA integrin tensions are also higher than 20 pN in this article. Therefore these tension sensors may not be able to differentially report the integrin tensions inside and outside FAs.

Previously we developed a tension sensor and modulator named TGT which has been applied to calibrate integrin tensions. TGT is a molecular linker synthesized from double-stranded DNA (dsDNA) with a programmable tension tolerance (*T*_tol_, tunable in 10 ~ 60 pN)^22^. Integrin ligand molecules are tethered through TGT on a surface where integrin tensions are globally restricted under *T*_tol_ because any tension larger than *T*_tol_ would rupture TGT and become abolished. Labeled with fluorophore, TGT rupture by integrin tensions causes local fluorescence loss on the surface which maps the spatial distribution of integrin tensions larger than *T*_tol_ in time-integrative manner. Note that the required force (*T*_tol_) to rupture dsDNAs was previously calibrated with a force loading time in the range of seconds^23^,^24^. The *T*_tol_ values of TGTs are nominal and subject to change if the integrin tension application time is much shorter or longer than seconds^24^. However, the monotonicity of *T*_tol_ values used in this article is expected to be unchanged. TGT has been applied to integrin tension measurement in FAs and revealed >54 pN tension in FAs^20^. Meanwhile we also found that cells produce a ~40 pN integrin tension during initial cell adhesion that happens before FA formation^20^. This tension might be the precursing outside-FA integrin tensions. However, the TGTs measuring the ~40 pN tension do not support cell stable adesion so no FAs can form on these surfaces, while the strong TGT supporting stable cell adhesion and focal adhesion formation can only measure the tension higher than 54 pN. As a result, outside-FA integrin tensions during stable cell adhesion can not be confirmed or calibrated by singleplex TGT.

Here we used multiplex tension gauge tether (mTGT) to overcome this dilemma. A surface coated with a mixture of TGTs at high *T*_tol_ (*T*_tol_ = 54 pN) value and low *T*_tol_ value allows cells to proceed to stable adhesion and enables the FA formation. Meanwhile, mTGT labeled with fluorophores at separate spectra simultaneously maps integrin tensions at multiple levels. Using mTGT, we confirmed, visualized and calibrated outside-FA integrin tensions, and compared their dynamics to integrin tensions in FAs. These two types of tensions are active in different tension ranges and respond independently to the inhibition of myosin, actin and microtubule, and serum deprivation. This discovery provides a more complete view about how integrins transmit cellular forces during stable cell adhesion.

## Results and Discussion

### Multiplex TGT simultaneously maps integrin tensions at multiple levels

**Figure 1:**
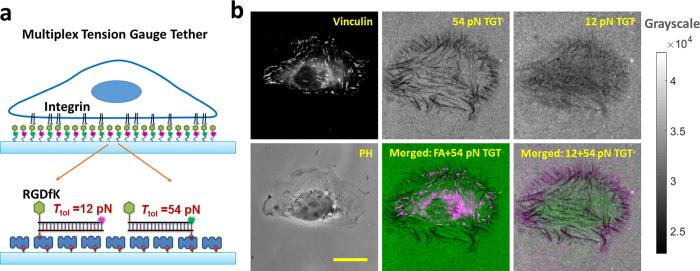
Integrin tensions outside focal adhesions (outside-FA integrin tensions) are confirmed and measured by multiplex TGT (mTGT). TGT rupture caused by integrin tensions produces fluorescence loss on the surface. The fluorescence loss in turn was used to map the spatial distributions of integrin molecular tensions. (**a**) TGTs with *T*_tol_ = 12 and 54 pN were labeled with Cy5 and Cy3 and applied to simultaneous mapping of integrin tensions at two levels (>12 and >54 pN) in adherent cells. (**b**) Phase contrast (PH) and fluorescence imaging of CHO-K1 cells on the mTGT surface. Focal adhesions (FAs) are visualized by vinculin immunostained with antibodies. 54 pN TGT rupture is in a streak pattern and 12 pN TGT rupture is homogeneously distributed at the cell-matrix interface. All FAs are located on the 54 pN TGT rupture tracks as shown in the merged image (FA and 54 pN TGT). The rupture patterns of 12 pN and 54 pN TGTs were merged and displayed in green and magenta colors, respectively. Scale bar: 10 μm.

To enable cell stable adhesion and simultaneous mapping of integrin tensions at multiple levels, we developed multiplex TGT (mTGT). Two types of TGTs with *T*_tol_ = 12 pN and 54 pN were synthesized from double-stranded DNA (dsDNA). These two TGTs were labeled with Cy5 and Cy3 respectively to map integrin tensions at different levels in separate imaging channels. These TGTs were conjugated at one end with peptide cyclic-RGDfK which is a ligand having high affinity with integrin α_V_β_3_^25^ and lower affinity with integrin α_5_β_1_^26^. The other end of the TGTs is tagged with biotin used to immobilize the TGTs on streptavidin coated surface. The TGTs with *T*_tol_ = 12 pN and 54 pN are in unzipping and shear configurations, respectively. In unzipping configuration, RGDfK and biotin are located at the same end of dsDNA with RGDfK conjugated to top strand and biotin conjugated to the bottom strand. In shear configuration, RGDfK and biotin are on the opposite ends of dsDNA, as shown in Fig 1a.

In experiments, a streptavidin-coated glass coverslip was incubated with mTGT, a mixture of 12 pN and 54 pN TGTs at 0.1 μM concentrations. The coverslip was then washed and served as an mTGT surface. CHO-K1 cells cultured for 72 hours in a culture flask were detached by EDTA solution (Ethylenediaminetetraacetic acid), a mild cell detaching reagent which preserves the integrity of integrins. Detached cells were plated on the mTGT surface and cultured for 2 hours in an incubator at 37 °C and 5% CO_2_. CHO-K1 cells adhered normally on the surface as 54 pN TGT has high *T*_tol_ value to support cell adhesion^22^. The mTGT surface was then imaged in three imaging channels: phase contrast, Cy3 and Cy5 (Fig. 1b). Significant TGT rupture occurred to 54 pN TGT-Cy3 and 12 pN TGT-Cy5. The 54 pN TGT rupture appears in a streak pattern. All FAs are located on the streak tracks of TGT rupture, demonstrating that clustered integrins in FAs transmit molecular tensions larger than 54 pN and rupture the TGT with *T*_tol_ = 54 pN (Fig. 1b and sFig. 1). FAs were visualized by immunostaining protein vinculin, a protein commonly used to mark FA locations. FA formation also demonstrated that cells adhered stably on the mTGT surface. In contrast to the streak pattern of 54 pN TGT rupture, the 12 pN TGT rupture is homogeneously distributed across the cell-substrate interface without being restricted in FAs, demonstrating that integrins outside FAs also transmit tensions with a force level larger than 12 pN. The homogeneous TGT rupture is insignificant on 54 pN TGT-Cy3, suggesting that integrin tensions out of FAs have a force level lower than 54 pN. Weak “bleed through” of 54 pN TGT rupture pattern was observed on 12 pN TGT image. This is reasonable because >54 pN integrin tensions in FAs are able to rupture 12 pN TGT. These two types of integrin tensions are further displayed in a merged image with green (12 pN TGT-Cy5) and magenta colors (54 pN TGT-Cy3) representing the spatial distributions of integrin tensions in the ranges of 12 ~ 54 pN and >54 pN, respectively (Fig. 1b). It is evident that two levels of integrin tensions have distinctly different spatial distributions with >54 pN tensions restricted in FAs and 12 ~ 54 pN tensions located outside FAs. The similar rupture patterns of two TGTs were also observed on an mTGT surface plated with other cell-line (MTC cells, Medullary thyroid carcinoma cell-line) as shown in sFig. 2. To rule out the possibility that the different patterns of fluorescence losses may be caused by the nature of Cy3 and Cy5 dyes, we synthesized 12 pN TGT-Cy3 and 54 pN TGT-Cy5 with swapping the fluorophores on the two sets of TGTs. TGT rupture patterns caused by cells on these two TGTs were acquired and compared to the results based on 12 pN TGT-Cy5 and 54 pN TGT-Cy3. The results show that TGT rupture patterns are independent of fluorophores and only determined by *T*_tol_ (sFig. 3). To rule out the possibility that the fluorescence loss in TGT rupture pattern may be caused by proteases or nucleases released by cells which digest TGT or streptavidin, as previous research reported such nuclease activity under cells^17^, we performed a control experiment by plating CHO-K1 cells on a surface coated with 54 pN TGT without RGDfK conjugation. This RGDfK-null TGT does not bind to integrins. The surface was also doped with RGDfK-biotin to support cell adhesion. After two hour incubation, cells adhere normally but no fluorescence loss was observed on the RGDfK-null TGT surface (sFig. 4), confirming that the fluorescence loss on regular TGT surfaces under CHO-K1 cells was caused by integrin tensions, not by nucleases or proteases.

Overall, mTGT simultaneouslly mapped integrin tensions at different level. We confirmed that the 54 pN TGT rupture is caused by integrins in FAs, in consistency with the previous report that motile FAs in CHO-K1 cells rupture 54 pN TGT^20^. The 12 ~ 54 pN integrin tension is clearly transmitted by integrins outside FAs and evenly distributed on the cell-substrate interface. Our experiments provide direct evidence that a substantial portion of integrin tensions exist outside FAs. To our knowledge, this is the first experiment visualizing outside-FA integrin tensions and quantifying their tension level in cells.

### Narrow down outside-FA integrin tension range with mTGT based on 12, 23, 33, 43 and 54 pN TGTs

We determined that outside-FA integrin tensions are in the range of 12 ~ 54 pN. To further narrow down the outside-FA integrin tension range and explore other possible tension levels with higher tension resolution, we prepared two series of TGTs with *T*_tol_ = 12, 23, 33, 43 and 54 pN (TGT structures shown in sFig. 5) and each group was labeled by Cy3 and Cy5 respectively. Note that intermediate *T*_tol_ values of 23, 33 and 43 pN, unlike 12 and 54 pN which were experimentally calibrated, were computed from dsDNA modeling and therefore the values are model-dependent. These values were predicted by de Gennes model with parameters derived from calibrated DNA shear force^22^. A recent improved model gives a new set of *T*_tol_ values of 16, 19 and 30 pN by taking experimental temperature and time scale into consideration^27^. Therefore, these intermediate *T*_tol_ values may deviate from true values and should be considered nominal in this article. However, the monotonic increase of *T*_tol_ as TGT geometry tuned from unzipping mode to shear mode should be unchanged.

First, we tested TGT rupture by cells on surfaces coated with singleplex TGTs. Because TGTs with *T*_tol_ = 12, 23 or 33 pN do not support cell adhesion^22^, RGDfK-biotin was doped on all TGT surfaces to assist cell adhesion. Streptavidin coated surfaces were incubated with a mixture solution of 0.1 μM TGT and 0.1 μM RGDfK-biotin for 30 min. CHO-K1 cells adhered normally on all five TGT surfaces doped with RGDfK-biotin. TGT rupture pattern is shown in sFig. 6. TGT rupture in both streak pattern (caused by integrins in FAs) and homogeneous pattern (caused by outside-FA integrins) are visible on 12, 23, 33 and 43 pN TGT surfaces. Homogeneous TGT rupture is less significant on 54 pN TGT surface. We quantified TGT rupture intensity caused by outside-FA integrins by specifically selecting the homogeneous fluorescence loss regions and analyzing the percentage of fluorescence loss in those regions. TGT rupture intensity caused by outside-FA integrins is 6.8% ~ 9.6% on 12 ~ 43 pN TGT surfaces and it significantly decreases to 2.8% on 54 pN TGT surface, suggesting that the peak of outside-FA integrin tension distribution is likely located in the range of 43 ~ 54 pN, or 30 ~ 54 pN if based on an improved dsDNA dissociation model^27^.

**Figure 2:**
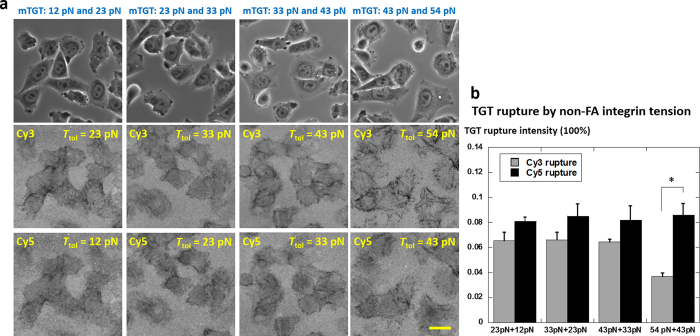
Analysis of TGT rupture caused by outside-FA integrin tensions. (**a**) TGT rupture patterns on a series of mTGT surfaces consisting of TGTs with adjacent *T*_tol_ values in 12, 23, 33, 43 and 54 pN. Scale bar: 20 μm. (**b**) TGT rupture intensities on homogeneous fluorescence loss regions (active regions of outside-FA integrins). Error bar represents standard error based on nine samples of cells.

Next, we tested whether there exist other distinguishable levels of integrin tensions in the range of 12 ~ 54 pN. We examined the difference of TGT rupture patterns and rupture intensities between two multiplex TGTs selected from the pool of 12, 23, 33, 43 and 54 pN TGTs. The TGT rupture images on four mTGT surfaces (12pN + 23pN, 23pN + 33 pN, 33pN + 43pN and 43pN + 54pN, labeled by Cy5 and Cy3 respectively) are presented in Fig. 2a. TGT rupture images of other mTGT combinations are included in sFig. 7. The TGT rupture intensities over the mTGT surface were analyzed and plotted in Fig. 2b. On the former three mTGT surfaces, TGT rupture patterns and rupture intensities have no significant difference. On the surface coated with mTGT of 43pN + 54pN, the TGT rupture intensity difference between two types of TGT caused by outside-FA integrin tensions is significant (*p* value = 0.048), suggesting that outside-FA integrin tensions rupture 12 ~ 43 pN TGTs at similar intensties but rupture much less 54 pN TGT. This result suggests that the majority of outside-FA integrin tensions are located in the range of 43 ~ 54 pN. We did not observe significant difference between two TGTs on other three mTGT surfaces in terms of either tension level or spatial distribution (Fig. 2b). Therefore no other distinguishable tension level was found by mTGT in the range of 12 ~ 43 pN.

### Temporal activities of outside-FA and FA integrin tensions

**Figure 3:**
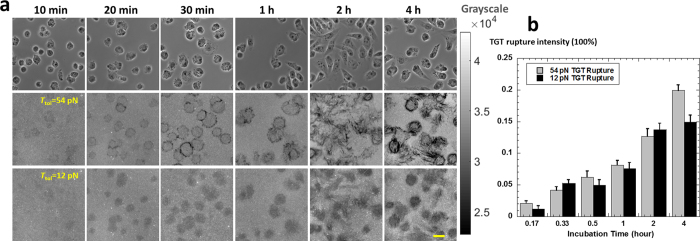
Temporal activities of outside-FA and FA integrin tensions. (**a**) 54 pN and 12 pN TGT rupture patterns were acquired in Cy3 and Cy5 imaging channels at a series of incubation times. Scale bar: 20 μm. (**b**) Both 54 pN and 12 pN rupture intensities increases by time. Error bar represents standard error based on nine cell samples.

With mTGT, we revealed that outside-FA integrin tensions coexist with FA integrin tensions in live cells. Next we monitored their temporal activities with a time series of experiments. CHO-K1 cells were plated on six mTGT surfaces (multiplexing of *T*_tol_ = 12 pN and 54 pN) and TGT rupture were imaged after incubation times of 10 min, 20 min, 30 min, 1 h, 2 h and 4 h, respectively (Fig. 3a). The results show that both 12 ~ 54 pN and >54 pN integrin tensions appeared as early as in 10 min after cell plating. From 10 min to 4 hours after cell plating, the 12 pN TGT rupture was consistently homogeneous underneath cells, indicating that 12 ~ 54 integrin tension was evenly distributed on the cell-substrate interface. 54 pN TGT rupture was distributed in the peripheral boundary of cells till 1 hours. At 2 and 4 hours, the 54 pN TGT rupture evolved to streak patterns as FAs are motile in CHO-K1 cells and rupture 54 pN TGT in their motion^20^. We quantified the TGT rupture and plotted the rupture intensities versus incubation times (Fig. 3b). Both 12 and 54 pN TGT rupture intensities continuously increase by time, suggesting that both outside-FA integrin tensions and integrin tensions in FAs are co-existing in cells. Even though FAs are generally formed in 1 hour after cell plating on our platform, outside-FA integrin tensions remain active in cells as the 12 pN TGT continued to be ruptured up to 4 hours which is the maximum incubation time we tested. Therefore, the formation of FAs did not eliminate the existence of outside-FA integrin tensions. The 12 pN and 54 pN TGT rupture intensities at each incubation time are at comparable levels, suggesting that the intensities of integrin tensions inside and outside FAs are also comparable. Therefore both types of integrin tensions should substantially contribute to the overall cellular forces. Note that the precise ratio of force contributions from these two types of integrin tensions is not equal to the ratio of TGT rupture intensities as TGT rupture efficiency by these integrin tensions are unknown. So we are unable to quantify the exact ratio of the contributions to overall cellular forces by the two types of integrin tensions. We also performed time-lapse imaging on an mTGT surface under one individual CHO-K1 cell. The cell was initially incubated for 1 hour until FAs were formed. 25 min time-lapse imaging shows that both streak rupture on 54 pN TGT and homogeneous rupture on 12 pN TGT consistently increased during the time-lapse imaging, confirming that outside-FA integrin tensions are active even after FA formation and both inside-FA and outside-FA integrin tensions coexist in cells during cell stable adhesion (sFig. 8).

### Dynamics of integrin tensions inside and outside FAs in response to various cell treatments

**Figure 4:**
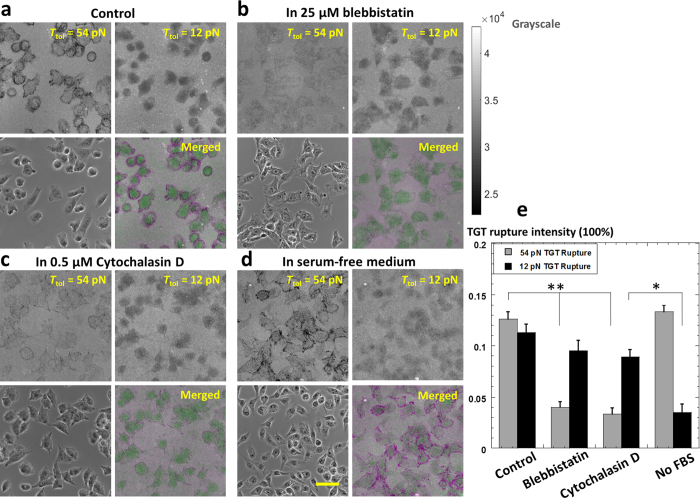
Dynamics of outside-FA and FA integrin tensions influenced by blebbistatin, cytochalasin D treatments and serum deprivation. (**a**) 12 pN and 54 pN TGT rupture by cells on the mTGT surface under normal cell culture conditions. (**b**) TGT rupture by cells with 25 μM blebbistatin treatment. (**c**) TGT rupture by cells with 0.5 μM cytochalasin D treatment. (**d**) TGT rupture by cells with serum deprivation. Scale bar: 50 μm. In the merged images, integrin tensions inside and outside FAs are represented by magenta and green colors, respectively. (**e**) Analysis of TGT rupture intensities. Error bar represents standard error based on nine cell samples.

We tested how dynamics of integrin tensions inside and outside FAs are affected by the treatments of blebbistatin and cytochalasin D which are commonly used to influence bulk cellular forces (Fig. 4). CHO-K1 cells on the mTGT surface coated with 12 and 54 pN TGTs were incubated for 2 hours in culture medium spiked with 25 μM blebbistatin which inhibits motor protein myosin II activity. TGT imaging shows that 54 pN TGT rupture intensity was 12.6% in the control sample and 4.0% in blebbistatin treated sample (Fig. 4a,b). The difference is significant with *p* value = 0.0078. In contrast, 12 pN TGT rupture was 11.3% in the control sample and 9.5% in blebbistatin treated sample without significant difference. Next we treated CHO-K1 cells on the mTGT surface with 0.5 μM cytochalasin D which disrupts actin polymerization. Under this treatment, 54 pN TGT rupture intensity was 3.3% which is significantly lower than the control result (*p* value = 0.0054) while 12 pN TGT rupture was 8.9% without significant difference from the control test (Fig. 4c). These results suggest that FA integrin tensions are highly sensitive to actin or myosin inhibitions, being consistent with the fact that actomyosin is the tension source for cellular forces transmitted by FAs^28^. However, actomyosin inhibition has much less influence on outside-FA integrin tensions, suggesting that outside-FA integrin tensions may originate from a tension source other than actomyosin. We also found that fetal bovine serum (FBS) addition in culture medium has strong influence on the activity of outside-FA integrin tensions (Fig. 4d). After cell incubation for 2 hours on mTGT surfaces, 12 pN TGT rupture by cells in serum-free medium is 3.5%, significantly lower than the 12 pN TGT rupture in the control sample with 10% FBS addition (*p* value = 0.0193), while the 54 pN TGT rupture has no significant difference from the control result, indicating that FBS deprivation causes little activity change of FA integrin tensions. This experiment demonstrated that FBS in culture medium upregulates the activity of outside-FA integrin tensions for unclear reasons. It is possible that the hormones and growth factors in the FBS may stimulate cells to exert outside-FA integrin tensions which in turn regulate normal cellular functions.

### Integrin tension distribution with microtubule formation inhibited in cells

**Figure 5:**
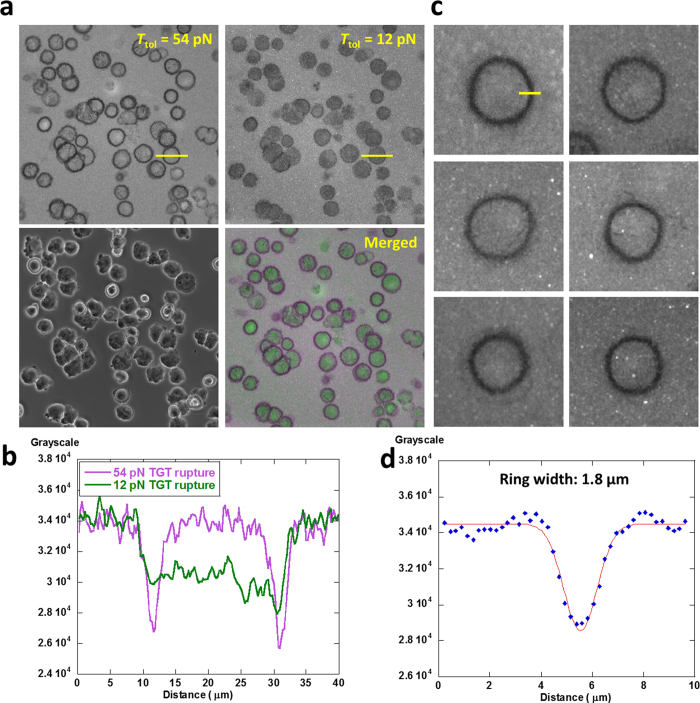
Microtubule inhibition perturbs the distribution of integrin tensions. (**a**) Distribution of integrin tensions in response to 1 μM nocodazole treatment. (**b**) Line profiles of 12 and 54 pN TGT rupture patterns, marked by yellow lines in Fig. 5a. (**c**) Representative 54 ~ 100 pN integrin tension distribution in ring patterns. (**d**) Line profile analysis on the ring patterns shows that the ring width is 1.8 μm in average.

Microtubule is one major component of cytoskeleton in mammalian cells. Its dynamics is important for cell migration and division^29^,^30^. Recent studies show that microtubule also regulates FA disassembly^31^, suggesting that microtubule may influence integrin tension distribution. Here we tested how microtubule disruption may change the dynamics of integrin tensions in cells. CHO-K1 cells in a culture flask were treated with 1 μM nocodazole which disrupts microtubule formation^32^,^33^ for 4 hours. Then the cells were detached and plated on an mTGT surface, coated with 12 pN and 54 pN TGTs in 1 μM nocodazole. After 2 hour incubation, cells adhered on the TGT surface but appeared in round shapes. Imaging of 12 pN and 54 pN TGT rupture revealed that 54 pN TGT rupture patterns by individual cells were all in ring shapes, indicating that >54 pN integrin tensions were concentrated in these ring areas (Fig. 5a,b). These rings are localized at the peripheral boundary of cells. The width of the rings is measured to be 1.8 μm in average as shown in Fig. 5c,d (optical resolution of fluorescence imaging is 0.3 μm under the imaging conditions used in this article). >54 pN integrin tensions were isotropically distributed in all directions of the rings. This may correlate with the low polarity of cells under microtubule inhibition as microtubule can induce and maintain cell polarity^34^. The outside-FA integrin tensions in the range of 12 ~ 54 pN are still homogeneously distributed under cells. TGT rupture analysis shows that 12 pN TGT rupture intensity (13.4%) is almost twice of 54 pN TGT rupture intensity (7.3%) averaged over cell adhesion area, indicating a higher percentage of outside-FA integrin tensions compared to normal cells. We concluded that microtubule inhibition significantly changes the distribution of >54 pN integrin tension, but has less impact on the distribution of outside-FA integrin tensions. The detailed mechanism for the spatial rearrangement of >54 pN integrin tensions is unclear and awaits further explorations. Nonetheless, this experiment demonstrated that the dynamics of integrin tensions are related to microtubule polymerization and mTGT is a powerful tool to monitor multiple levels of integrin tensions in terms of both tension level and spatial distribution.

## Conclusions

We developed mTGT to co-map multiple levels of integrin tensions and explored the integrin tensions outside FAs. mTGT overcomes the obstacle that the TGT required for measuring integrin tensions at a low level may not support cell adhesion. On an mTGT surface, cells stably adhered with FA formation and integrin tensions were simultaneously mapped at different levels. mTGT revealed that integrins outside FAs also transmit tensions which were mainly located in the range of 43 ~ 54 pN. These outside-FA tensions are dispersedly distributed at the cell-substrate interface and coexist with integrin tensions inside FAs. Both types of tensions substantially contribute to the bulk cellular forces. As FAs have been viewed as the primary sites transmitting cellular forces^5^, mTGT reveals new insights into integrin-transmitted cellular forces and provides a feasible approach for the study of outside-FA integrin tensions.

Compared to other contemporary integrin tension sensors, mTGT has a broader tension detection range of 10 ~ 60 pN which covers the active range of integrin tensions. On an mTGT surface, tension signals represented by TGT fluorescence loss are acquired in a time-integrative manner which increases signal intensity and improves signal-to-noise ratio of the integrin tension map. This feature enables the observation of outside-FA integrin tensions with a low surface density of tension. mTGT also provides a convenient platform to monitor the change of integrin tension profile in response to drug treatments. We monitored integrin tensions in FAs and outside FAs and found that they have different responses to the treatments of blebbistatin, cytochalasin D, nocodazole and serum deprivation. mTGT can be potentially applied to the efficacy test of drugs that alter mechanical properties and mechano-sensing abilities of cells. For instance, metastatic and non-metastatic cancer cells might have different cellular force characteristics as metastatic cancer cells adjust their mechanical properties to adapt to the surrounding microenvironment^35^. This may lead to the different dynamics of integrin tensions from normal cells. mTGT coated surfaces can be conveniently integrated with microfluidics and applied to chemical screening to search for the drugs which correct the integrin tension profile in metastatic cells. Overall, mTGT provides a convenient and robust platform to comprehensively monitor the spatial distribution and dynamics of integrin tensions.

## Materials and Methods

### TGT Synthesis

The TGT is built on dsDNA conjugated with RGDfK peptide ligand. Cyclic peptide RGDfK targeting integrin α_V_β_3_ was conjugated to single-stranded DNA at the 3′ end. The 5′ end of the ssDNA is labeled with a fluorophore such as Cy3, Cy5 or Atto 488. This ssDNA conjugated with fluorophore and RGDfK is annealed with the complementary ssDNA with a biotin tag which determines the tension tolerance of annealed dsDNA. ssDNAs were purchased from Integrated DNA technologies, Inc. The sequence and modification of the top strand are shown below:

5′-/5Cy3/GGC CCG CAG CGA CCA CCC/3ThioMC3-D/-3′

Cyclic peptide RGDfK-NH_2_ (catalog #: PCI-3696-PI) was purchased from Peptides International, Inc. RGDfK and the top strand were conjugated together through hetero-bifunctional crosslinker Sulfo-SMCC (22622, Thermo Fisher Scientific Inc.). Sulfo-SMCC has maleimide and NHS ester groups on two ends which react with the thiol group on thiol-modified DNA and amine on RGDfK, respectively. The ssDNA-RGDfK conjugate was further purified by ethanol precipitation.

Sequence and structure for 12 pN and 54 pN TGTs

12 pN TGT:

5′-/5Cy5/GGC CCG CAG CGA CCA CCC /RGDfK/-3′

3′-CCG GGC GTC GCT GGT GGG /Biotin/-5′

54 pN TGT:

5′-/5Cy3/GGC CCG CAG CGA CCA CCC /RGDfK/-3′

3′-/Biotin/CCG GGC GTC GCT GGT GGG-5′

The structures of TGTs with Ttol = 23, 33 and 43 pN are shown in sFig. 5.

### Preparation of mTGT Surfaces

All TGTs were immobilized on a glass surface through streptavidin-biotin bonds. Glass bottom petridish (D35-14-1.5-N, *In Vitro* Scientific) was incubated with 1 mg/ml BSA-biotin (Bovine serum albumin, A8549, Sigma-Aldrich) in PBS for 1 hour, and then the surface was washed by PBS solution 3 times. BSA-biotin provides biotin tags for streptavidin coating and BSA also suppresses non-specific cell adhesion. The surface was then incubated with 200 μg/ml streptavidin for 30 min. 12 pN TGT-Cy5 and 54 pN TGT-Cy3 or other combinations were mixed at concentration of 0.1 μM and 0.1 μM, respectively. Multiplex TGT solution was incubated on the streptavidin surface for 30 min at 4 °C. Finally, the surface was washed by PBS for three times without drying. Cell solution was loaded on the TGT surface immediately after the washing procedure.

### Mapping integrin tensions on mTGT surfaces

#### CHO-K1 cell culture

CHO-K1 cell was cultured with F-12 K Medium (Kaighh’s Modification of Ham’s F-12 Medium, Cat. No.30–2004), with 10% FBS, 1% streptomycin/penicillin in a standard cell culture incubator with 5% CO_2_ at 37 °C. The cells were passaged every three days with a dilution of 1:30 and used at around 70 to 90% confluence prior to mTGT assays.

#### Cell seeding on mTGT surfaces

CHO-K1 cells were detached by EDTA solution [100 mL 10 X HBSS + 10 mL 1 M HEPES (PH7.6) + 10 mL 7.5% sodium bicarbonate + 2.4 mL 500 mM EDTA +1 L H_2_O] for 10 minutes, the harvested cells was centrifuged at 300 g for 3 min and resuspended in F-12 K Medium with FBS at a seeding concentration of 10^6^ cells/ml. After plating cells on mTGT surfaces, they were incubated in a cell culture incubator for 2 hours or other appropriate times based on experimental design.

#### Observation of fluorescence loss due to TGT rupture caused by integrin forces

CHO-K1 cells were plated and incubated at 10^6^/ml density on 12 ~ 54 pN TGT surfaces at 37 °C and 5% CO_2_ for 2 hours or other required times. The cell samples were then relocated onto a multi-channel fluorescent microscope (Nikon Ti E) enclosed in a 37 °C thermal chamber. Images were acquired using 40× objective. In some experiments, cells were fixed after 2 hour incubation on mTGT surface and immunostained with antibody against vinculin (FAK100, Millipore) and secondary antibody (goat anti-mouse IgG labeled with FITC, AP124F, Millipore).

#### Quantification of TGT rupture

The TGT rupture marked by fluorescence loss under individual cells was quantified by matlab. The grayscale fluorescent images of mTGT surfaces were imported into matlab. Individual cell regions were manually selected and the average fluorescence intensity (grayscale) in the cell region was calculated. The percentage of fluorescence loss in these regions was computed using the equation:

Fluorescence loss ratio = (I_Background_ − I_Cell region_)/I_Background_, where “I” stands for fluorescence intensity in the grayscale images.

During the analysis of TGT rupture by FAs on 54 pN TGT surface, the entire cell regions were selected for the fluorescence loss analysis. During the analysis of TGT rupture by outside-FA integrin tensions, only the homogeneous fluorescence loss regions underneath cells were selected.

## Additional Information

**How to cite this article**: Wang, Y. and Wang, X. Integrins outside focal adhesions transmit tensions during stable cell adhesion. *Sci. Rep.* **6**, 36959; doi: 10.1038/srep36959 (2016).

**Publisher’s note**: Springer Nature remains neutral with regard to jurisdictional claims in published maps and institutional affiliations.

## Supplementary Material

Supplementary Information
