# Supplementary material for: Integrins outside focal adhesions transmit tensions during stable cell adhesion
Source: Sci Rep. 2016 Nov 15;6:36959. doi: 10.1038/srep36959 (PMC5109487; doi:10.1038/srep36959)
Supplement: Supplementary Information [file srep36959-s1.pdf]

Integrins outside focal adhesions transmit tensions during stable cell adhesion

Yongliang Wang[1] and Xuefeng Wang[1,2*]
[1]Department of Physics and Astronomy, Iowa State University, 12 Physics Hall, Ames, IA 50011, USA.
[2]Molecular, Cellular, and Developmental Biology interdepartmental program, Molecular Biology Building, Ames, IA 50011, USA.
Correspondence and requests for materials should be addressed to X.W. (xuefeng@iastate.edu)

Supplementary Figures

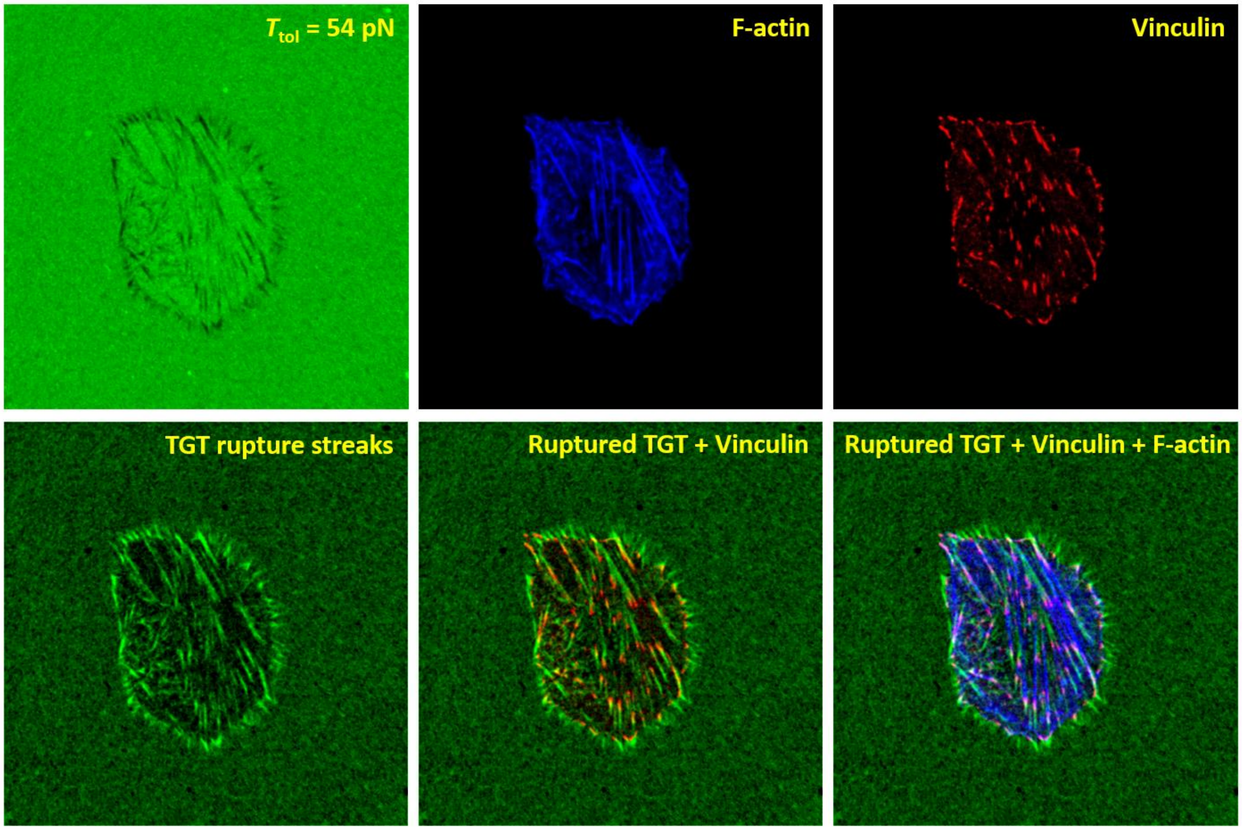

sFig. 1. Co-localization of TGT rupture and focal adhesions. Every TGT rupture streak ends with a focal adhesion (marked by immunostained vinculin), demonstrating that the streak pattern of 54 pN TGT rupture is caused by integrin tensions in motile focal adhesions in CHO-K1 cells.

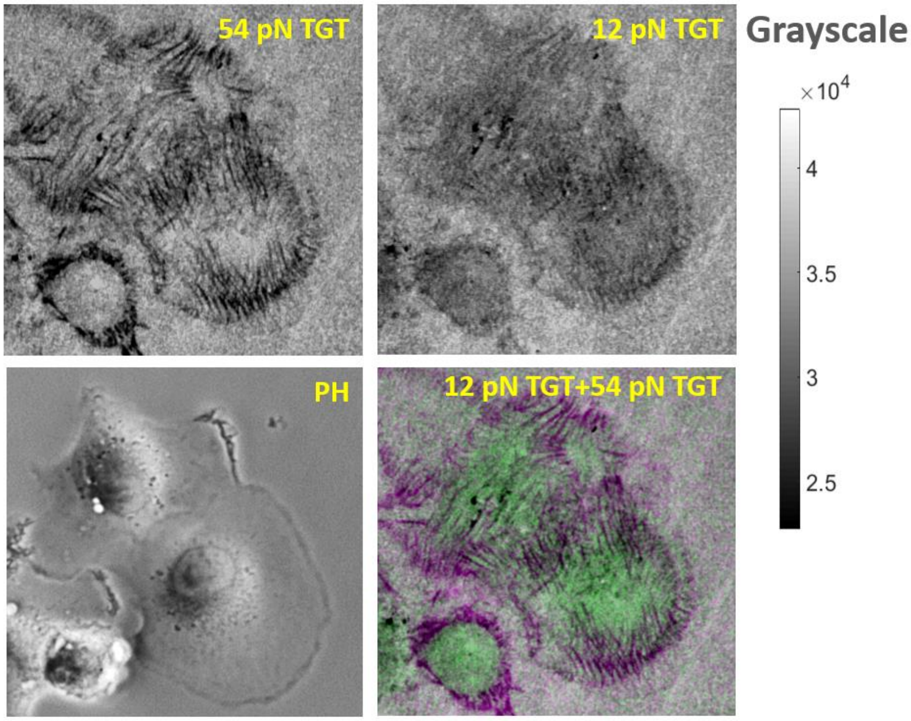

sFig. 2. On mTGT surface, streak rupture pattern of 54 pN TGT and homogeneous rupture pattern of 12 pN TGT were also observed to be caused by another cell-line: MTC cells (Medullary thyroid carcinoma cell-line).

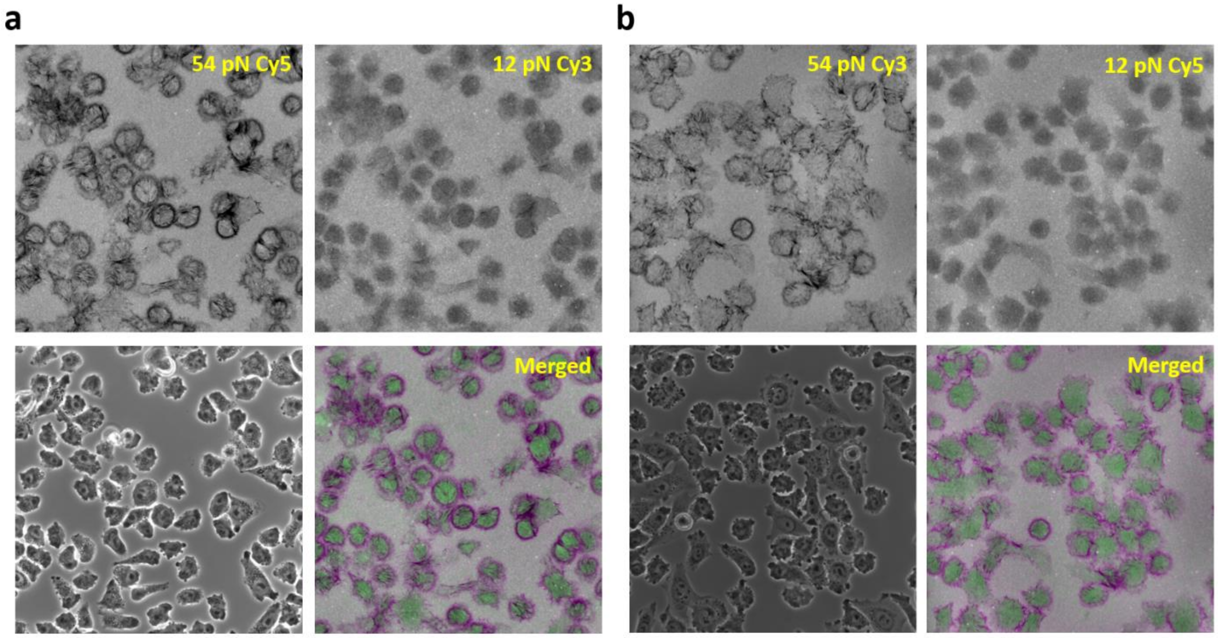

sFig. 3. Switching Cy3 and Cy5 dyes on 12 pN and 54 pN TGTs. In the merged images, magenta and green are used to represent the 54 pN and 12 pN TGT rupture patterns, respectively, regardless of the labeled fluorophores. (a) TGT rupture pattern on the surface coated with Cy5-labeled 54 pN TGT and Cy3-labeled 12 pN TGT. (b) TGT rupture pattern on the surface coated with Cy3-labeled 54 pN TGT and Cy5-labeled 12 pN TGT. The 54 pN TGT rupture in both (a) and (b) was in streak pattern, and the 12 pN TGT rupture in both (a) and (b) was homogenous under cells. Therefore, fluorophores on TGTs did not influence TGT rupture pattern which is only determined by tension tolerance of the TGT.

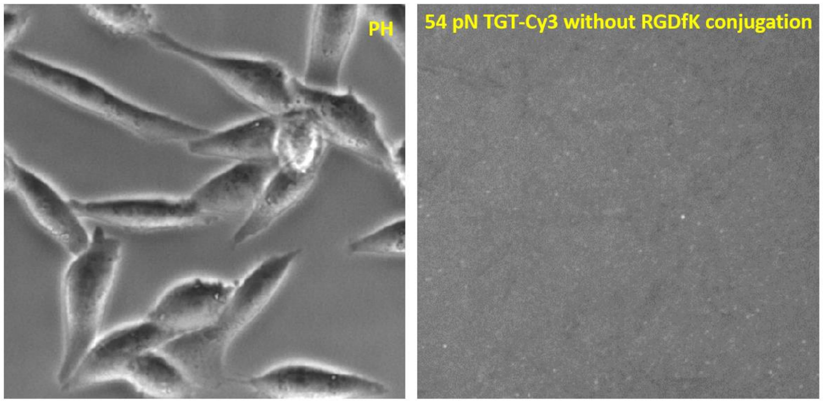

sFig. 4. No fluorescence loss was observed on a surface coated 54 pN TGT without RGDfK conjugation, confirming that the fluorescence loss on a regular TGT surface is caused by integrin tensions in the experiments in this article, not by other potential factors such as nucleases or proteases. The surface was doped with RGDfK-biotin to support cell normal adhesion.

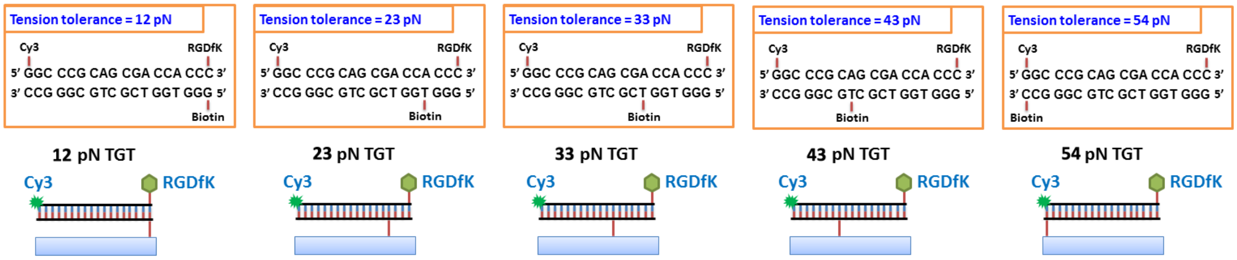

sFig. 5. dsDNA sequence and Structures of 12~54 pN TGTs.

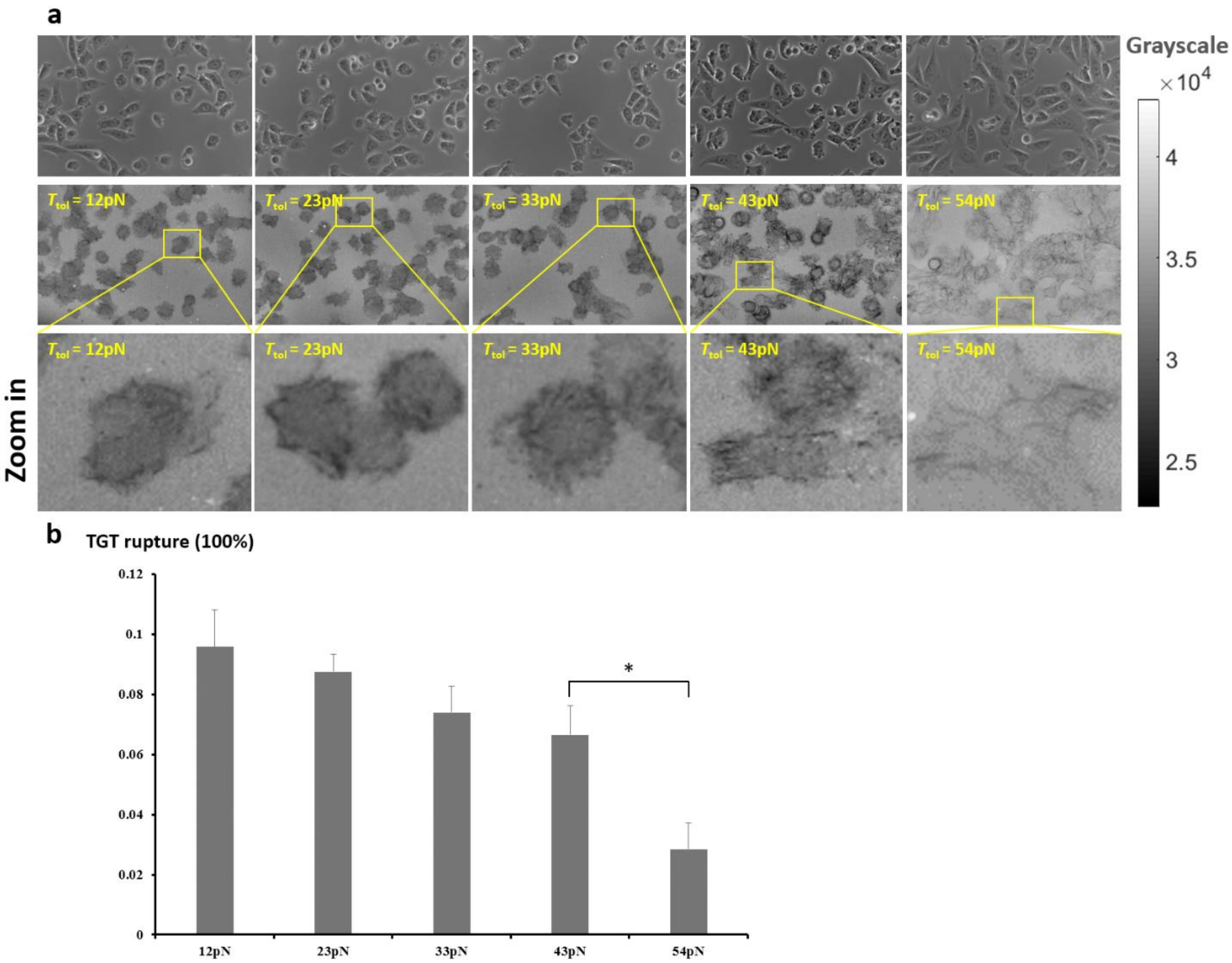

sFig. 6. TGT rupture by CHO-K1 cells. (a) Each TGT of 12~54 pN was mixed with RGDfK-biotin with 1:1 ratio of molar concentration at 0.1 µM final concentration. RGDfK-biotin enables CHO-K1 cell adhesion on all TGT surfaces including 12~33 pN TGTs. TGT ruptures by both FA integrin tensions and non-FA integrin tensions are visible on 12, 23 and 33 pN TGT surfaces. (b) TGT rupture by non-FA integrin tensions. The rupture analysis was performed on homogenous fluorescence loss region by avoiding streak rupture caused by FA integrin tensions. A T_{tol} threshold was observed between 43 pN and 54 pN, indicating the non-FA integrin tensions has a peak distribution in 43~54 pN. CHO-K1 cells were incubated on the surfaces for 2 hours before imaging.

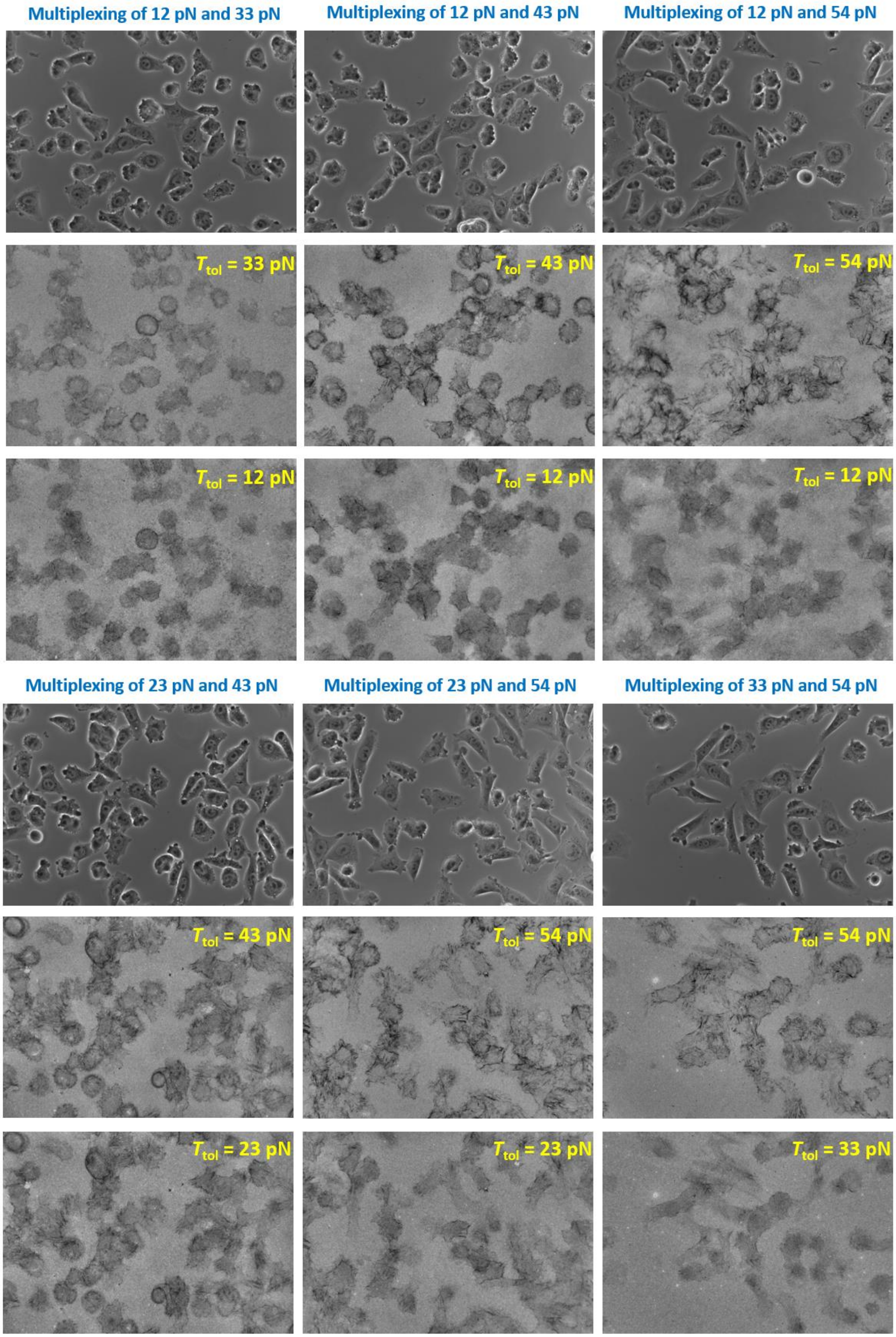
Multiplexing of 12 pN and 33 pN
Multiplexing of 12 pN and 43 pN
Multiplexing of 12 pN and 54 pN
T_{tol} = 33 pN
T_{tol} = 43 pN
T_{tol} = 54 pN
T_{tol} = 12 pN
T_{tol} = 12 pN
T_{tol} = 12 pN
Multiplexing of 23 pN and 43 pN
Multiplexing of 23 pN and 54 pN
Multiplexing of 33 pN and 54 pN
T_{tol} = 43 pN
T_{tol} = 54 pN
T_{tol} = 54 pN
T_{tol} = 23 pN
T_{tol} = 23 pN
T_{tol} = 33 pN

sFig. 7. TGT rupture on the surfaces coated with mTGTs at various combinations of T_{tol}s. Each surface was coated with the mixture of mTGT (two TGTs) and RGDfK-biotin at 0.1 µM final concentration. CHO-K1 cells were incubated on the surfaces for 2 hours before imaging.

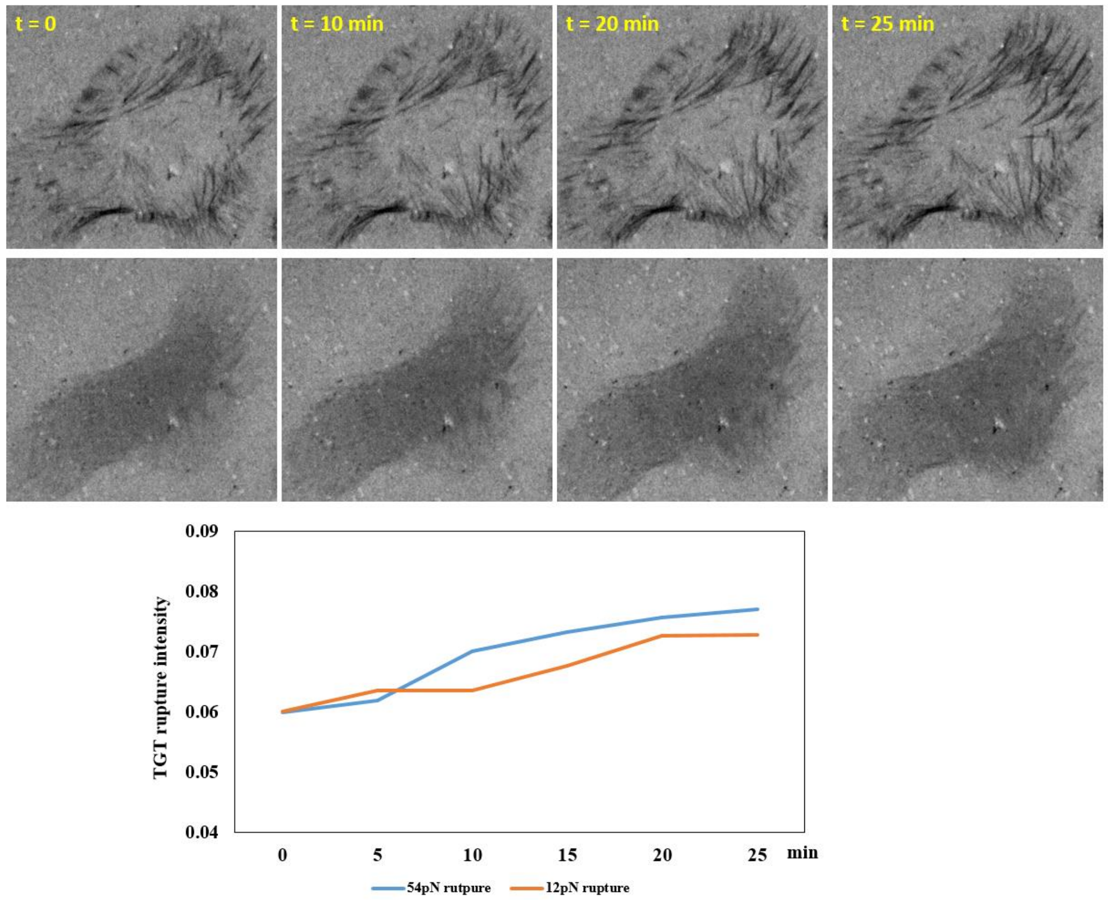

sFig. 8. Time-lapse imaging of TGT rupture by an individual CHO-K1 cell on an mTGT surface coated with 54 pN TGT-Cy3 and 12 pN TGT-Cy5. The imaging began after cell incubation on the surface for 1 hour. The rupture of both TGTs increased gradually by time.
